# Risk of developing psychiatric disease among adult patients with skin disease: A 9‐year national register follow‐up study in Norway

**DOI:** 10.1002/ski2.294

**Published:** 2023-10-20

**Authors:** Flora Balieva, Dawit Shawel Abebe, Florence J. Dalgard, Lars Lien

**Affiliations:** ^1^ Department of Dermatology Stavanger University Hospital Stavanger Norway; ^2^ Faculty of Health Sciences University of Stavanger Stavanger Norway; ^3^ Norwegian National Advisory Unit on Concurrent Substance Abuse and Mental Health Disorders Innlandet Hospital Trust Brumunddal Norway; ^4^ Department of Nursing and Health Promotion Oslo Metropolitan University Oslo Norway; ^5^ Division of Mental Health and Addiction Vestfold Hospital Trust Tønsberg Norway; ^6^ Department of Dermatology and Venereology Skåne University Hospital University of Lund Malmö Sweden; ^7^ Faculty of Social and Health Sciences Inland Norway University of Applied Sciences Elverum Norway

## Abstract

**Background:**

The existing association between skin disease and psychiatric comorbidity has gained attention during the last decades. Stress and mental illness can directly or indirectly affect skin disease, while dermatological conditions, known to impair life quality and mental well‐being, can promote psychiatric conditions.

**Objectives:**

The aim of this study was to assess the risk of developing psychiatric disease among adult dermatological patients over a period of time. The secondary objective was to see which psychiatric disorders developed most commonly, and which skin diseases posed the greatest risk for later mental health issues.

**Methods:**

Adult dermatological patients were followed for 9 years (2008–2016) using the Norwegian Patient Registry, for both outpatient and inpatient specialist healthcare services. Dermatological patients were identified during the first 2 years and were then followed for psychiatric comorbidity prospectively for the next 7 years.

Cox regression models were applied to estimate the risks of psychiatric disorders among patients with skin diseases. Estimates were adjusted for age and gender differences. Hazard risk ratios (HR) with 95% CI are reported.

**Results:**

Dermatological patients developed depressive disorders most frequently (4.1% vs. 2.3% in non‐dermatological participants), followed by anxiety disorders (3.3% vs. 1.8%), and adjustment disorders (2.6% vs. 1.5%). Developing depressive disorders showed the highest HR among dermatological patients, HR (95% CI) = 2.5 (2.4–2.5), followed by disorders related to alcohol use, HR (95% CI) = 2.2 (2.1–2.5), and anxiety disorders, HR (95% CI) = 2.1 (2.1–2.2). Papulosquamous disorders were the skin conditions with the highest HR for developing a mental health condition, with depressive disorder having HR (95% CI) = 2.6 (2.5–2.9); anxiety disorders at HR (95% CI) = 2.9 (2.7–3.1); and disorders related to alcohol use at HR (95% CI) = 3.2 (2.8–3.6).

**Conclusions:**

The study demonstrates that having a skin disease doubles to triples the risk of developing a psychiatric illness within 7 years, especially depression, anxiety, and alcohol use compared with the general population.

## Graphical abstract

Skin disease and psychiatric comorbidity have gained attention during the last decades. We aimed to assess the risk of developing psychiatric disease among adults with an existing dermatological condition over a 9‐year period and report Hazard risk ratios adjusted for age and gender. The secondary objective was to see which psychiatric disorders developed most commonly, and which skin diseases posed the greatest risk for later mental health issues. Depression developed most frequently, thereafter anxiety and adjustment disorders. Papulosquamous disorders were the skin conditions with the highest HR for developing a mental health disorder, with HR for depression at 2.6; anxiety at 2.9; and alcohol use at 3.2.

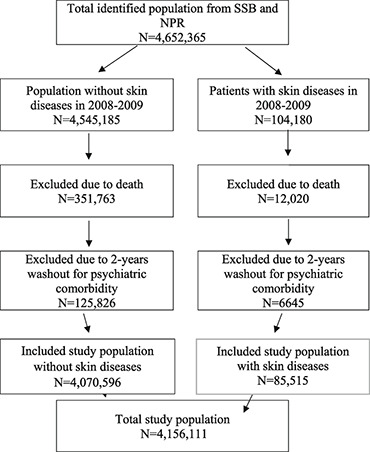

1

**What is already known about this topic?**

- The significant association between skin disease and psychiatric comorbidity has been demonstrated in cross‐sectional studies.

**What does this study add?**

- This follow up study over 9 years demonstrated that patients with common skin diseases have a 2–3 times higher risk to develop psychiatric comorbidities.

## 2 INTRODUCTION

The significant association between skin disease and psychiatric comorbidity has been highlighted during the last decades in cross‐sectional study designs.^1^, ^2^, ^3^ A recent multi‐centre study among dermatological patients across Europe demonstrated that depression, anxiety, and body dysmorphia are more common in patients with skin diseases and lead to a reduced health related quality of life (HRQoL).^4^, ^5^ Depression, anxiety and suicidal ideation are considered to be among the most common mental diseases that can directly affect skin disease,^6^ but other psychiatric conditions are not thoroughly enough explored to make conclusions for psychiatric disorders as a whole. Nevertheless, other primary, less frequent psychiatric conditions among dermatological patients gain more attention: obsessive‐compulsive disorder (OCD) involving the skin, trichotillomania, skin picking disease, acne excoriée, dermatitis artefacta and delusion of parasitosis are among the more frequently encountered psychocutaneous disorders.^7^ Body dysmorphic disorder and delusions may lead to self‐mutilation of the skin.^8^

Psychiatric conditions can also affect skin disease indirectly.^9^, ^10^, ^11^ Adverse effects of psychotropic drugs may cause dermatological side effects. Alcohol abuse is known to be more prevalent in inflammatory skin conditions.^12^ Depression and anxiety may be a reason for noncompliance with the dermatological therapy,^6^ not meeting for appointments and treatments.^11^ Substance abuse is known to predispose for skin disease as well as lead to negligence, lack of self‐care and exposure to the elements causing or complicating a skin condition.^7^

Itch, pain, fatigue, scratching, avoidant coping and stigmatisation due to a skin condition are known to impair quality of life and mental well‐being, which reduces HRQoL and in turn can promote psychiatric disease.^13^, ^14^, ^15^, ^16^, ^17^, ^18^, ^19^, ^20^ Therapies for skin diseases may be more burdensome than conventional treatments for other somatic conditions, and specific dermatologic therapies can further reduce HRQoL^21^ by being time‐consuming, messy, painful or complicated. Side effects of dermatological systemic therapy may likewise contribute to psychiatric morbidity.^9^

Some psychiatric conditions are more thoroughly explored in context of their association to skin disease (depression, anxiety),^1^, ^2^, ^3^ while others are explored to a lesser extent (autism, eating disorders,^22^ adjustment disorders^23^) and association, directionality or bidirectionality between skin conditions and these less‐explored psychiatric conditions can only be speculated at this point.

Even when an association between two events is established in cross‐sectional study design, the bidirectionality doesn't readily allow for conclusions on causality because of the complex biopsychosocial interactions. Longitudinal studies are thus highly needed, performed on a large population to better understand how patterns in somatic disease, may increase the risk of psychiatric comorbidity. This study gives the opportunity to explore a broad range of psychiatric conditions that develop in dermatological patients.

The aim of the present study was to assess the risk of developing psychiatric disease among adults with skin diseases over time. Moreover, we aimed to investigate which psychiatric disorders developed most commonly and which skin diseases posed the greatest risk for later mental health issues.

## 3 PATIENTS AND METHODS

### 3.1 Study design and population

**FIGURE 1:**
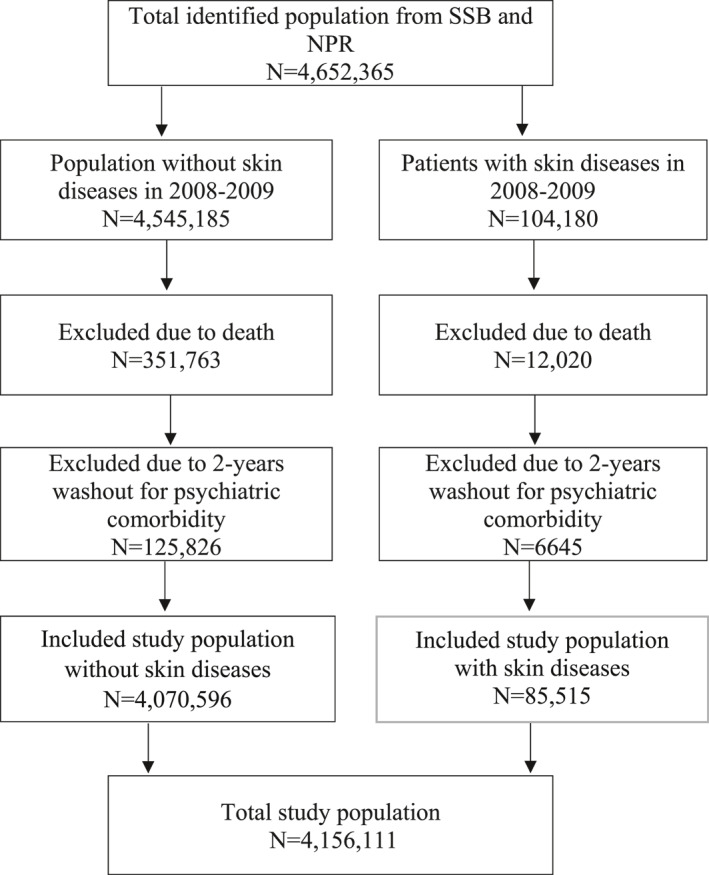
Study population flow chart. **SSB:** Statistisk Sentralbyrå (Statistics Norway); **NPR:** Norwegian Patient Register.

This is a national register‐based cohort study combining sociodemographic information from Statistics Norway and information on somatic diseases and mental disorders obtained from the Norwegian Patient Registry. Norwegian Patient Registry holds data on all registered diagnoses obtained during contacts with specialist health care services. A unique number is assigned to each person, which enables to generate health statistics without concern of duplicates of dermatological or psychiatric events and makes it possible to track the same patient over time and from hospital to hospital. All diagnoses are based on the background of International Classification of Diseases 10^th^ Revision (ICD‐10) codes.^24^ As illustrated in Figure 1, the study population consisted of adults, 18 years or older, who were legal residents in Norway as of 1 January 2008 to 31 December 2016 (*N* = 4 652 365). Individuals with specific skin diseases (*N* = 104 180, 2.2%; see Table 1 for the list of skin diseases) were identified during the preceding 2 years (2008–2009), and then followed for psychiatric comorbidity for 7 years, from 1 January 2010, through 31 December 2016. Patients who were registered as deceased (*N* = 363 783) during the study period (2008–2016) were excluded from analysis. Moreover, we included a two‐year washout period for event variables (2008–2009) and excluded all individuals who developed a psychiatric disease during the washout period.

We included the most common psychiatric diagnoses and all dermatological conditions with the exception of actinic keratosis and skin cancers. These are non‐inflammatory conditions, previously shown to have a low and uncertain association with mental health.^25^, ^26^ Also, the number of patients with bullous disorders were low and results are not presented.

Disorders of skin appendages (L60–L75) include disorders of the nails, hair (alopecias), sweat glands (hyperhidrosis) and sebaceous glands (acne, rosacea, hidradenittis suppurativa (HS)). Papulosquamous disorders (L40 – L45) include Psoriasis, parapsoriasis, pityriasis rosea, lichen planus and pityriasis rubra pilaris. Dermatitis and Eczemas. Pruritus (L20‐L30) include atopic eczema, contact and allergic dermatitis, pruritus and other specified eczemas. Urticaria and Erythema (L50‐L54) include all forms of urticaria, as well as erythema multiforme, nodosum, and other specified erythemas. Infections of the skin (L00‐L08) exclude venereal infections and those that are not specifically a cause of a skin infection (non‐L codes).

### 3.2 Exposure, outcome and covariate variables

All diagnoses were acquired during outpatient and inpatient contacts with specialist healthcare between 2008 and 2016. We defined skin and psychiatric diseases according to the ICD‐10 codes.^24^ Dichotomous variables are given in Table 1 presenting ICD‐10 diagnostic categories coded to specific disorders.

**TABLE 1:** ICD‐10 codes and year of diagnosis for primary exposure and outcome variables.

	ICD‐10 codes	Year diagnosis was identified
**Primary exposure variables (Dermatological diseases)**		
Infections of the skin and subcutaneous tissue	L00‐L08	2008–2009
Dermatitis and eczema. Pruritus	L20‐L30	2008–2009
Papulosquamous disorders	L40‐L45	2008–2009
Urticaria and erythema	L50‐L54	2008–2009
Disorders of skin appendages	L60‐L75	2008–2009
**Outcome variables (Psychiatric diseases)**	**ICD‐10 codes**	**Year of diagnosis**
Disorders related to alcohol use	F10	2010–2016
Depressive disorder	F32‐F34	2010–2016
Anxiety disorders	F40‐F41	2010–2016
Post‐traumatic stress disorder (PTSD)	F43.1	2010–2016
Obsessive‐compulsive disorder (OCD)	F42	2010–2016
Adjustment disorders	F43.2	2010–2016
Somatoform disorders	F45	2010–2016
Eating disorders	F50	2010–2016

### 3.3 Covariates

Age, gender and socio‐economic status (SES) were used as covariate variables. Age, as of 1 January 2008, was used as a continuous variable. Socio‐economic status was included as a moderator variable and as a covariate variable. We included being recipient of social benefits as an index for SES.

### 3.4 Statistical analysis

The Cox proportional regression models were applied to estimate the risks of psychiatric diseases (event outcomes) among dermatological patients (an independent risk factor). Hazard ratios (HRs) with 95% confidence intervals (95% CIs) were reported, with calendar year as the underlying time axis. Hazard risk ratios (HR) estimates were adjusted for age, gender and recipient of social welfare (SES indicator). We tested the proportional hazard assumptions on the basis of Schoenfeld residuals after fitting each model (“*estat phtest tests”*) and found that some covariates did not meet the proportional hazards assumption. Thus, we added interaction terms between time and time‐variant covariates (age and SES), and specified stratification for gender. Estimates were judged as statistically significant when *p*‐values<0.05. The analyses were performed using Stata SE/17.

## 4 RESULTS

The diagnostic ICD‐10 codes for the dermatological and psychiatric conditions included in this study can be seen in Table 1.

In Table 2, descriptive summary for age, gender and SES and prevalence of psychiatric diseases among persons with and without a skin disease are presented. Results show that depression was the most frequent mental disorder that occurred during the 7‐year period in both patients with and without a skin disease, but much more frequently (4.1%) in dermatological patients than in non‐dermatological individuals (2.3%).

**TABLE 2:** Descriptives for individuals with and without a skin disease.

	Persons with a skin disease (*N* = 85 515)	Persons without a skin disease (*N* = 4,192,433)
Age in years: mean (±SD)	45.9 (±16.4)	45.5 (±16.2)
Gender		
Male N (%)	39 555 (1.7%)	2,321,699 (98.3%)
Female N (%)	45 960 (2.6%)	1,748,872 (97.4%)
Recipient of social welfare: mean (±SD)	0.07 (±0.4)	0.04 (±0.3)
Disorders related to alcohol use	1125 (1.3%)	33 856 (0.8%)
Depressive disorders	3531 (4.1%)	94 030 (2.3%)
Anxiety disorders	2838 (3.3%)	74 828 (1.8%)
Post‐traumatic stress disorder	653 (0.8%)	17 088 (0.4%)
Obsessive compulsive disorder	228 (0.3%)	6085 (0.2%)
Adjustment disorders	2223 (2.6%)	60 157 (1.5%)
Somatoform disorders	584 (0.7%)	12 412 (0.3%)
Eating disorders	250 (0.3%)	5148 (0.1%)

*Note*: Summary for age and recipients of social welfare: mean (±SD). Frequencies (N, row %) for gender and psychiatric diseases developing among individuals with and without previously only skin diseases during a 7‐year period (2010–2016).

Table 3 shows that disorders of the skin appendages (nail, hair and sebaceous/apocrine/eccrine gland disorders, represented by diseases such as HS, alopecias, acne, rosacea, hyperhidrosis) were the ones where new‐onset depression (5.5%) was highest. Anxiety was the second most frequent psychiatric disorder among dermatological patients with 3.3% versus 1.8% in patients without a skin disease. The highest anxiety was seen among patients with infections of the skin and subcutaneous tissue (4.6%) and disorders of skin appendages (4.5%). Next were adjustment disorders 2.5% versus 1.5%, highest among patients with urticaria and erythema (3.8%). Infectious diseases showed the highest frequency for developing disorders related to alcohol use (2.9%) and Post‐Traumatic Stress Disorder (PTSD) (1.6%). For all other mental health disorders, the frequency was around or below 1%.

**TABLE 3:** Frequencies (row %) of psychiatric diseases developing among patients with previously only skin diseases during a 7‐year period (2010–2016).

	Disorders related to alcohol use *N* = 34 981	Depressive disorders *N* = 97 561	Anxiety disorders *N* = 77 666	Post‐traumatic stress disorder *N* = 17 741	Obsessive compulsive disorder *N* = 6313	Adjustment disorders *N* = 62 380	Somatoform disorders *N* = 12 996	Eating disorders *N* = 5398
Infections of the skin and subcutaneous tissue^a^ (N 19125)	554 (2.9%)	986 (5.2%)	874 (4.6%)	315 (1.6%)	71 (0.4%)	665 (3.5%)	136 (0.7%)	90 (0.5%)
Dermatitis and eczema. Pruritus^b^ (*N* = 27706)	348 (1.3%)	1251 (4.6%)	1024 (3.7%)	242 (0.9%)	108 (0.4%)	818 (2.9%)	33 (0.8%)	101 (0.4%)
Papulosquamous disorders^c^ (*N* = 25758)	383 (1.5%)	892 (3.5%)	782 (3.1%)	184 (0.7%)	62 (0.2%)	604 (2.4%)	153 (0.6%)	51 (0.2%)
Urticaria and erythema^d^ (*N* = 4342)	59 (1.3%)	219 (5.2%)	182 (4.2%)	44 (1.0%)	16 (0.4%)	165 (3.8%)	50 (1.1%)	20 (0.5%)
Disorders of skin appendages^e^ (*N* = 21920)	312 (1.4%)	1167 (5.5%)	960 (4.5%)	261 (1.2%)	97 (0.4%)	729 (3.4%)	184 (0.8%)	100 (0.5%)

^a^ Excluding venereal infections and infections non‐L diagnoses (L00‐L08).

^b^ Atopic eczema, contact and allergic dermatitis, pruritus and other specified eczemas (L20‐L30).

^c^ Psoriasis, parapsoriasis, pityriasis rosea, lichen planus and pityriasis rubra pilaris (L40‐L45).

^d^ All forms of urticaria, as well as erythema multiforme, nodosum, and other specified erythemas (L50‐L54).

^e^ Nail, hair, sebaceous and sweat gland diseases, acne, rosacea, HS (L60‐L75).

The results from the cox regression models are presented in Table 4, where regression estimates (HR) were adjusted for age, gender, and SES. Hazard risk ratios for developing any mental disorder were statistically significant for all dermatological conditions. The highest HRs (95% CI) were for developing alcohol use disorders at 3.2 (2.8–3.6), *p* < 0.001, in patients with papulosquamous diseases. Papulosquamous disorders (diseases such as psoriasis, parapsoriasis and lichen planus) had the highest HR for all mental health disorders except for somatoform disorders, where dermatitis, eczemas and pruritus showed highest HR (95% CI) = 1.5 (1.1–1.9), *p* < 0.01. Eating disorders did not develop at a higher ratio for any of the skin conditions.

**TABLE 4:** Risk of developing psychiatric diseases among patients with skin diseases compared to the general population presented as hazard risk ratios (HR) and 95% Confidence Interval (95% CI) from 2010 to 2016.

Exposure variables	Event outcomes							
	Disorders related to alcohol use	Depressive disorders	Anxiety disorders	Post‐traumatic stress disorder (PTSD)	Obsessive‐compulsive disorder (OCD)	Adjustment disorders	Somatoform disorders	Eating disorders
	HR (95% CI)	HR (95% CI)	HR (95% CI)	HR (95% CI)	HR (95% CI)	HR (95% CI)	HR (95% CI)	HR (95% CI)
All skin diseases	2.2 (2.1–2.4)***	2.5 (2.4–2.5)***	2.1 (2.1–2.2)***	1.1 (1.0–1.2)*	1.2 (1.0–1.3)*	1.1 (1.0–1.1)*	1.2 (1.1–1.4)**	1.1 (0.9–1.2)
Infections of the skin and subcutaneous tissue^a^	2.6 (2.4–2.9)***	2.2 (2.1–2.4)***	2.2 (2.1–2.4)***	0.9 (0.8–1.1)	0.8 (0.6–1.1)	0.9 (0.8–1.1)	1.1 (0.8–1.5)	0.9 (0.6–1.2)
Dermatitis and eczema. Pruritus^b^	1.9 (1.7–2.1)***	2.1 (1.9–2.1)***	2.1 (1.9–2.2)***	1.1 (0.9–1.3)	1.6 (1.3–2.1)***	1.2 (1.0–1.3)*	1.5 (1.1–1.9)**	1.0 (0.8–1.3)
Papulosquamous disorders^c^	3.2 (2.8–3.6)***	2.6 (2.5–2.9)***	2.9 (2.7–3.1)***	1.1 (0.9–1.4)	1.1 (0.8–1.6)	1.1 (0.9–1.2)	1.0 (0.7–1.5)	0.9 (0.7–1.4)
Urticaria and erythema^d^	1.5 (1.4–2.4)***	2.1 (1.8–2.4)***	2.1 (1.8–2.4)***	0.9 (0.6–1.5)	0.9 (0.5–1.8)	1.3 (1.0–1.7)*	0.4 (0.1–1.2)	1.0 (0.6–1.8)
Disorders of skin appendages^e^	1.7 (1.2–2.3)**	1.9 (1.8–2.1)***	1.8 (1.7–1.9)***	1.3 (1.1–1.5)***	1.2 (0.9–1.6)	1.1 (1.0–1.3)*	1.3 (0.9–1.7)	1.1 (0.9–1.4)

*Note*: Estimates adjusted for age, gender, and recipient of social welfare (SES indicator).

^a^ Excluding venereal infections and infections non‐L diagnoses (L00‐L08).

^b^ Atopic eczema, contact and allergic dermatitis, pruritus and other specified eczemas (L20‐L30).

^c^ Psoriasis, parapsoriasis, pityriasis rosea, lichen planus and pityriasis rubra pilaris (L40‐L45).

^d^ All forms of urticaria, as well as erythema multiforme, nodosum, and other specified erythemas (L50‐L54).

^e^ Nail, hair, sebaceous and sweat gland diseases, acne, rosacea, HS (L60‐L75).

*p‐values < 0.05; **p‐values < 0.01; ***p‐values < 0.001.

## 5 DISCUSSION

The main finding from this study is that having a skin disease doubles to triples the risk of developing any mental health disorder within 7 years compared with the rest of the population.

These results confirm earlier cross‐sectional studies that report depression and anxiety to be significantly prevalent among patients with skin diseases across Europe.^4^ Disorders related to alcohol use in our study were likewise seen to hold a significantly higher risk. For these three psychiatric disorders (depression, anxiety and alcohol use), the dermatological patients as a whole had a more than doubled risk of developing them regardless of the diagnostic group (*p* < 0.01).

In this study, HRs for depression, anxiety and alcohol use were more than 2‐fold for the whole study group, and more than 2‐fold for each dermatological group, except ‘Disorders of skin appendages’ where HRs were just below 2‐fold.

Patients with disorders of skin appendages (ICD‐10 L60‐L75) like acne, rosacea and HS showed a 90% higher risk for depression (HR 1.9; 95% CI (1.8–2.1, *p* < 0.001) and 80% higher risk for anxiety 1.8 (1.7–1.9, *p* < 0.001). Other studies have examined new‐onset depression and anxiety in skin patients, specifically rosacea, alopecia and HS. Incidence rates per 1000 person‐years were examined and calculated by Egeberg et al.^27^ and showed that rosacea was associated with a severity‐dependent, increased risk of depression and anxiety disorders, similar to our study, around 2‐fold.

Another study on a disorder of skin appendages, alopecia areata (AA), performed by Vallerand et al., attempted to assess bidirectionality between AA and major depressive disorder using a UK registry.^28^ The authors found that AA increased the risk of subsequently developing major depressive disorder by 34% (HR 1.34; 95% CI, 1.23–1.46; *P* < 0.001). Similar findings between AA and new‐onset depression and anxiety were found in another UK study.^29^

The cohort study by Therlacius^30^ revealed that HS showed an increased risk of completed suicide after adjustment for confounding factors (HR 2.42; 95% CI, 1.07–5.45) and an increased risk of antidepressant drug use (HR 1.30; 95% CI 1.17–1.45). The risk of drug use was lower than for completed suicide, therefore one could speculate if these patients were underdiagnosed and undertreated for an existing depression, an established risk factor for suicide. This may suggest that risk for depression may be even higher than assumed. Underdiagnosing depression in dermatological patients was also observed in another study from the same year, 2018.^31^ Together with our findings, disorders of skin appendages, regardless of the specific diagnosis, increase the risk of depression and anxiety around two‐fold.

In this study, papulosquamous disorders showed HR 2.6 for depression, 2.9 for anxiety and 3.2 for diseases related to alcohol use (Table 4). Among the papulosquamous disorders, psoriasis is most common and has a high prevalence in Norway.^32^ Oh et al.^33^ showed that psoriasis patients were at an increased risk for depression, anxiety and somatoform disorders compared with their referent cohort, but patients with moderate‐to‐severe psoriasis had a higher risk than patients with mild disease. Hazard ratios for psoriasis patients in Oh et al.'s study ranged between 1.21 and 1.60 for the three psychiatric categories. In our study this risk was much higher. Of notice, the latter study was performed on a Korean population. Cultural differences may have played a role, but regardless of the differences in degree of risk the results of both studies highlight the burden of psoriasis and psoriasiform disorders (papulosquamous group). The risk of depression, anxiety, and suicidality in patients with psoriasis from a cohort study by Kurd et al.^18^ likewise showed the risk to be increased, but again with lower HRs than in our study (just above 30% for depression and anxiety), however patients with more severe psoriasis had a more than 70% higher risk.

A study on the risk of developing psychiatric disorders in paediatric patients with psoriasis found a higher risk^22^ for depression, in accord with our study. On the contrary, our study revealed that the risk of alcohol use in patients with papulosquamous diseases was higher, which was not seen in the Kimball et al.'s study. This difference is probably due to the paediatric population in the latter.^22^

Patients with psoriasis had approximately a 60% greater risk of dying due to alcohol‐related causes compared with peers of the same age and sex in the general population in the study by Parisi et al.^34^ During a median (IQR) of 4.4 (6.2) years of follow‐up, the alcohol‐related mortality rate was 4.8 per 10 000 person‐years for the psoriasis cohort, versus 2.5 per 10 000 for the comparison cohort.

Although the risks of developing certain psychological conditions in papulosquamous disorders vary somewhat between studies, there is a clear heightened risk for developing several psychiatric conditions in patients with papulosquamous diseases.

We documented that HR is more than doubled for developing depression and anxiety in patients with eczemas and pruritus. Schonman et al.^35^ identified 526 808 adults with atopic eczema who were matched to 2 569 030 without. Atopic eczema was associated with an increased incidence of new‐onset depression and anxiety, and the risk for depression increased with atopic eczema severity. This was less true for new‐onset anxiety. The study concluded that adults with atopic eczema are more likely to develop new depression and anxiety. Our study also concludes this, but the risks for anxiety and depression for eczemas and pruritus were much higher than the risk Schonman et al. found in their study, even in his severe cases of eczema.

In our study, we saw no risk of developing eating disorders in the dermatological population as a whole, and for the separate dermatological conditions. The only cohort study to examine ED was the study by Kimbal et al.^22^ and they also did not see a higher risk for this disorder. ED develops in young age and most adult dermatological patients might already be suffering from an ED at the identification point and been excluded. The only conclusion that can be made is that no study so far has seen a higher risk for ED in dermatological patients, whether adults or children.

Only patients with eczemas and pruritus among our dermatological population showed a higher risk for developing a somatoform disease HR (95% CI) = 1.5 (1.1–1.9), *p* < 0.01. None of the other skin diseases were at higher risk. Pruritic conditions force patients to constantly focus on and engage in scratching behaviour, perhaps predisposing to developing a somatoform disorder.

Adjustment disorders' risk of developing was higher at *p* < 0.05 significance level only for eczemas and pruritus; urticaria and erythema; and disorders of skin appendages. No other cohort studies were found in the literature.^23^ To truly explore any risk, newer, larger studies need to be performed, for any conclusions to be made with certainty.

For OCD only eczemas and pruritus showed to be at higher risk, with a significance at the 0.001 level. No other cohort studies exploring development of OCD in eczemas and pruritus exist, but our study shows that this area needs to be thoroughly explored to evaluate this high risk. The same can be said about the risk of developing PTSD, seen to be significantly higher, *p* < 0.001 in diseases of the skin appendages. If earlier and better treatment of these dermatological conditions were made more available, further studies could explore if PTSD could be reduced in these patients.

### 5.1 Strengths and limitations:

To our knowledge this is the first large longitudinal study examining the risk of dermatological patients developing a broad range of common psychiatric disorders. Data were collected from registers avoiding selection bias, however, only adult individuals with inflammatory skin conditions seen by a specialist were included, which limits the generalisability for the whole Norwegian population. The values we present may be an underestimation, since patients attending general practitioners or undiagnosed for either a dermatological or a psychiatric disorder are not included. The association between skin diseases and developing a psychiatric disorder is complex, and associations may be influenced by factors such as comorbidities, smoking status, body mass index, physical activity, among others. In registry‐based studies, such data may be unavailable.

Although studies investigating HR of dermatological patients developing depression and anxiety in diseases like psoriasis and eczema exist, the present study adds to the literature of rarer psychiatric disease categories and some other dermatological conditions.

## 6 CONCLUSION

This large follow‐up study suggests that skin diseases may double to triple the risk of developing a psychiatric illness within 7 years especially regarding depression, anxiety and alcohol use compared with the rest of the population. Since many skin diseases are chronic, the management of dermatological patients should include screening for psychiatric suffering and offer adequate care when needed. Results can be utilised for policy development in the allocation of special services for the target groups, such as developing psychodermatological units, including teams with dermatologists and psychiatrists as installed in several hospitals across Europe.^36^ Future research should continue to examine this relationship to help develop holistic treatment approaches.

## AUTHOR CONTRIBUTIONS

**Flora Balieva**: Conceptualisation (supporting); Formal analysis (supporting); Funding acquisition (supporting); Investigation (lead); Methodology (supporting); Project administration (supporting); Supervision (lead); Validation (lead); Visualisation (lead); Writing – original draft (lead); Writing – review & editing (lead). **Dawit Shawel Abebe**: Conceptualisation (supporting); Data curation (lead); Formal analysis (lead); Funding acquisition (lead); Investigation (lead); Methodology (lead); Project administration (supporting); Supervision (supporting); Validation (supporting); Visualisation (supporting); Writing – original draft (supporting); Writing – review & editing (equal). **Florence Jorgensen Dalgard**: Conceptualisation (equal); Formal analysis (supporting); Investigation (supporting); Methodology (supporting); Project administration (supporting); Supervision (supporting); Validation (supporting); Writing – original draft (supporting); Writing – review & editing (equal). **Lars Lien**: Conceptualisation (equal); Funding acquisition (lead); Methodology (supporting); Project administration (lead); Supervision (supporting); Validation (supporting); Visualisation (supporting); Writing – original draft (supporting); Writing – review & editing (equal).

## CONFLICT OF INTEREST STATEMENT

None to declare.

## ETHICS STATEMENT

All study procedures were approved by the Norwegian Regional Committee for Medical and Health Research. Informed Consent to participate was not required since this study uses already existing administrative data and was waived by the Norwegian Regional Committee for Medical and Health Research Ethics South East Norway (ref: 17/26919‐5). All authors declare that all procedures contributing to this work have been performed in accordance with the ethical standards of the 1975 Helsinki Declaration. All registry owners approved the use of their data.

## Data Availability

The data that support the findings of this study are available from Statistics Norway and Norwegian Directorate of Health for the Norwegian Patient Register, but restrictions apply to the availability of these data, which were used under license for the current study, and so are not publicly available. Data are however available from the corresponding author upon reasonable request and with permission of Statistics Norway and Norwegian Directorate of Health for the Norwegian Patient Register.
